# Investigation into Influence of Tensile Properties When Varying Print Settings of 3D-Printed Polylactic Acid Parts: Numerical Model and Validation

**DOI:** 10.3390/polym16162253

**Published:** 2024-08-08

**Authors:** Khalil Homrani, Steven Volcher, Edouard Riviere Lorphèvre, Anthonin Demarbaix, Jérémy Odent, Margaux Lorenzoni, Laurent Spitaels, François Ducobu

**Affiliations:** 1 Machine Design and Production Engineering Lab, UMONS Research Institute for Materials Science and Engineering, University of Mons, Place du Parc 20, 7000 Mons, Belgium; 2 Science and Technology Research Unit, Science and Technology Department, Haute Ecole Provinciale de Hainaut (HEPH), Condorcet University Square Hiernaux 2, 6000 Charleroi, Belgium; 3 Laboratory of Polymeric and Composite Materials (LPCM), Center of Innovation and Research in Materials and Polymers (CIRMAP), University of Mons, Place du Parc 20, 7000 Mons, Belgium

**Keywords:** additive manufacturing, polymer, material extrusion, fused deposition modelling, numerical modeling, tensile testing, factorial design of experiments

## Abstract

Material Extrusion (MEX), particularly Fused Filament Fabrication (FFF), is the most widespread among the additive manufacturing (AM) technologies. To further its development, understanding the influence of the various printing parameters on the manufactured parts is required. The effects of varying the infill percentage, the number of layers of the top and bottom surfaces and the number of layers of the side surfaces on the tensile properties of the printed parts were studied by using a full factorial design. The tensile test results allowed a direct comparison of each of the three parameters’ influence on the tensile properties of the parts to be conducted. Yield strength appears to be the most affected by the number of layers of the top and bottom surfaces, which has twice the impact of the number of layers of the side surfaces, which is already twice as impactful as the infill percentage. Young’s modulus is the most influenced by the number of layers of the top and bottom surfaces, then by the infill percentage and finally by the number of layers of the side surfaces. Two mathematical models were considered in this work. The first one was a polynomial model, which allowed the yield strength to be calculated as a function of the three parameters mentioned previously. The coefficients of this model were obtained by performing tensile tests on nine groups of printed samples, each with different printing parameters. Each group consisted of three samples. A second simplified model was devised, replacing the numbers of layers on the side and top/bottom surfaces with their fractions of the cross-section surface area of the specimen. This model provided results with a better correlation with the experimental results. Further tests inside and outside the parameter ranges initially chosen for the model were performed. The experimental results aligned well with the predictions and made it possible to assess the accuracy of the model, indicating the latter to be sufficient and reliable. The accuracy of the model was assessed through the R2 value obtained, R^2^ = 92.47%. This was improved to R^2^ = 97.32% when discarding material infill as an input parameter.

## 1. Introduction

The application of additive manufacturing (AM) in rapid machining and manufacturing has the ability to bring to life the complex components of computer designs [1]. However, due to the limitations of pure-polymer printing in terms of mechanical properties and performance, it is important to understand the impact and effects of the variation in all types of printing parameters [2].

**Figure 1:**
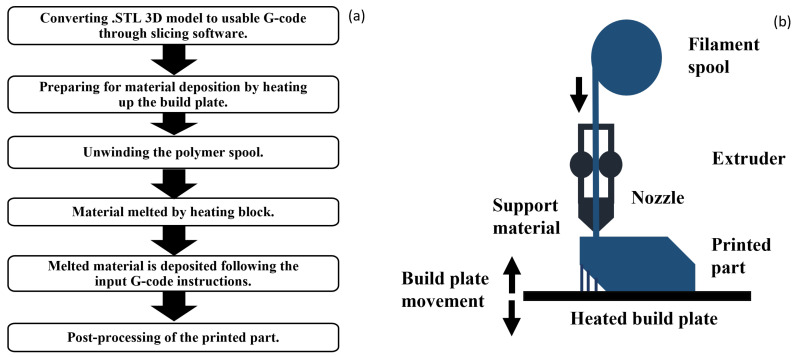
Fused Filament Fabrication (FFF) printing steps (**a**) and diagram (**b**).

The ISO/ASTM 52900:2021 standard [3] defines seven classes or categories of AM technologies, known as “process families”. These categories were classified based on the primary materials and processes employed. Figure 1a illustrates the typical steps followed when parts are printed with Fused Filament Fabrication (FFF) technology [4]. Figure 1b illustrates a schematic depicting Fused Filament Fabrication (FFF). In particular, the AM stages involve the use of the STL file format, which offers several advantages, including portability [5], simplicity [6] and being the most widely used format [7]. However, it occasionally encounters issues related to geometry discontinuity [8].

FFF falls under the category of Material Extrusion (MEX) [9]. MEX encompasses several processes wherein the material is selectively dispensed through a nozzle or orifice to construct parts layer by layer. Specifically, FFF begins with polymer materials in a spool of filament subjected to melting via a specialized device. The liquefied material is then meticulously deposited layer by layer through a nozzle of known diameter, recreating the input CAD model provided in STL format [10]. The horizontal movement of the nozzle follows a predefined set of instructions compiled from the STL format by using slicing software [11]. This movement facilitates the controlled deposition of the melted material onto the build platform, often referred to as a heated bed. Subsequently, the deposited material gradually cools down and solidifies layer by layer [12,13]. To allow solidification to occur and to prevent the part from peeling from the build platform, the temperature of the bed is maintained below the extrusion temperature [14,15]. Subsequently, post-processing methods and adjustments to the geometry are implemented to attain the desired final shape [11].

Polylactic acid (PLA) is the most widely used 3D-printing material due to its biodegradable, bio-sourced and inexpensive nature and acceptable mechanical properties [16]. It can be printed at lower temperatures, between 180 and 220 °C, compared with acrylonitrile butadiene styrene’s (ABS) 240–270 °C range [17] or polyethylene terephthalate glycol’s (PETG) 220–260 °C range [18]. It is, however, more fragile, while ABS is shock- and impact-resistant. ABS emits strong fumes when melted and requires a heated plate to prevent deformation, as mentioned above, during printing [19]. PETG is a compromise between PLA and ABS, as it has more impact resistance and flexibility than PLA but is easier to print than ABS, although it is easier to damage afterwards. It can withstand a temperature of 80 °C, which is between those of PLA, 60 °C, and ABS, 105 °C.

**Figure 2:**
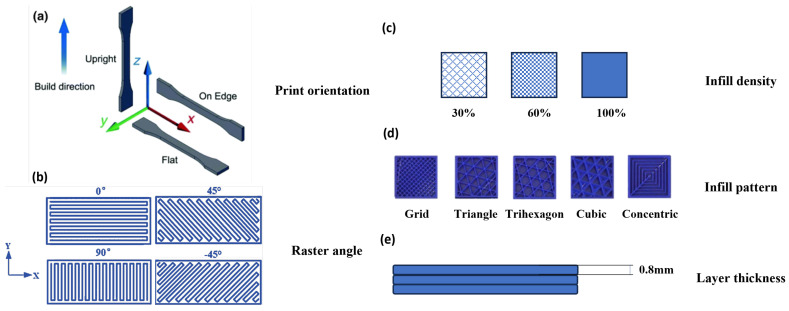
Explanation of various printing parameters for FFF-printed parts: build direction (**a**), raster angle (**b**), infill percentage (**c**), infill pattern (**d**) and layer thickness (**e**).

A key mechanical characteristic frequently explored in additive manufacturing (AM) is yield strength [20]. This property denotes the maximum tensile stress a material can endure before failure or fracture. It plays a pivotal role in assessing the structural soundness and load-bearing capability of printed objects. Gaining insights into the variables affecting yield strength is vital to refining the printing process and attaining the desired properties in the final product. To investigate this, research was previously conducted on the yield strength of 3D-printed ASTM-D638 test specimens, which were utilized in multiple studies, each focusing on different printing parameters, as shown in Figure 2.

Srinivasan et al. [21] and Srinivasan et al. [22] focused on PETG-printed parts with a general look at various printing parameters, namely, infill percentage, layer thickness, raster angle, and build orientation. Similarly, Srinivasan et al. [23] drew a comparison among the various commonly used infill patterns for the parts. Gonabadi et al. [24] worked with PLA samples produced through FFF to test the effects of build orientation and infill pattern/density on their tensile properties. This study proved to be the most well rounded in providing a general understanding of the effects of printing parameters on the tensile properties of printed parts and was chosen to serve as experimental validation for this work. Non-standard geometry studies such as Yadav et al.’s [25] and Weake et al.’s [26] utilized novel geometries to test the impact of varying similar print settings on the tensile properties of FFF parts, while in most cases, the samples are subjected to a standardized, well-defined and repeatable yield strength test. Rodriguez et al. [27], Casavola et al. [28] and Rankouhi et al. [29] noted that optimal mechanical properties are attained when the filament deposition direction aligns with the tensile load direction. All three studies came to similar conclusions about the important impact of deposition direction on yield strength. Other studies focusing on layer height and raster orientation are those by Lanzotti et al. [30] and Ziemian et al. [31], where the impact of these parameters on the tensile properties of 3D-printed PLA and ABS parts was studied; the exact correlation denotes that certain raster orientations increase the yield strength by up to 50% for PLA parts and 44% for ABS parts. These findings suggest that increasing the raster angle to 90° from the vertical axis of the specimen diminishes yield strength, whereas lower layer heights enhance it. Investigating into the effect of layer thickness and air gap, Durgun et al. [32] and Chacon et al. [33] revealed that negative air gap and minimal layer thickness can enhance the mechanical properties of ABS-printed parts.

The impact of these various printing parameters on the mechanical properties of 3D-printed materials can be assessed by establishing numerical models. A common type of numerical model is regression [34], which is in essence a tool that allows for predicting the mechanical properties of 3D-printed components based on print setting data. Regression models use statistical techniques to establish relationships between input variables, such as print speed, layer height, and temperature, and the resulting mechanical properties, like tensile strength. By analyzing experimental data, regression models can identify how changes in print settings impact mechanical performance, enabling accurate predictions and the optimization of printing parameters. These models can range from simple linear regressions to more complex forms, such as polynomial or multiple regressions, depending on the complexity of the relationships between variables [35]. The data necessary for the creation of such models have been extensively collected in various works by using experimental methodologies such as the Taguchi method, full factorial design and Response Surface Methodology. An example study incorporating these methods is Nyiranzeyimana et al.’s [36], which utilized Digimat additive manufacturing 2020 software and the Taguchi experimental design to predict how printing temperature, layer thickness and print speed influence residual stresses. The study achieves near convergence between simulations and experiments, with a 3.7% error. The mechanical properties of 3D-printed carbon fiber composite products were within acceptable ranges: tensile strength, compressive strength, Young’s modulus and elongation were all within the expected values. These findings provide a practical approach to FDM, reducing trial and error and enhancing manufacturing capabilities. Similarly, Zhao et al. [37] developed bio-compatible femur bone structures by using FFF with a medical-grade polymer composite. A new test specimen was designed based on an X-ray micro-CT scan of a femur bone. The experimental characterization employed a cascade approach, including a factorial experimental design, where all possible combinations of factor levels were tested, allowing the study to assess not only the individual impact of each factor but also how factors interacted with each other for screening and identifying key process parameters, and a Taguchi design for process optimization. Additionally, a computational Finite Element Analysis (FEA) model was used to explore the underlying physical phenomena observed in the experiments. Similarly, Anoop et al. [38] utilized Response Surface Methodology (RSM) and investigated how infill density, layer height and raster orientations affect the elastic behavior of FDM components. They created a predictive model using microscale Representative Volume Elements (RVEs) based on the 0/90° raster configuration to estimate elastic properties. Their study revealed that for directional behavior, especially with higher layer heights at lower infill densities, the development of an RSM-based model to optimize these traits for functional applications proved helpful in quantifying the impact of print settings on the mechanical properties of the parts.

The primary objective of this study is to assess the impact of three selected parameters on the mechanical properties of FFF-printed parts. We establish a regression model from experimental data, through which a unique numerical investigation is conducted. We explore the effect of material infill [39], the number of layers of top and bottom surfaces and the number of layers of side surfaces on the mechanical properties of printed parts. Furthermore, within the scope of this paper, the overarching goal is to develop a predictive model capable of anticipating the tensile properties of parts, calculated directly from their print settings. The findings generated will serve as a valuable resource for guiding the design and manufacturing processes of 3D-printed parts across various applications.

## 2. Experimental Validation and Methodology

### 2.1. Material Properties and Equipment Calibration

The experimental protocol begins with the printing of samples in an attempt at reproducing results from the literature. This is an important step to verify that the experimental procedure is valid. A first test was carried out to determine whether it was possible to reproduce the results from the experimental validation study [24]. That paper was chosen due to its comprehensive coverage of various 3D printing parameters. It meticulously explores these parameters, offering a thorough understanding of their impact on the tensile properties of the printed part. Moreover, the paper provides detailed experimental results, and the recreation details provided are precise, enabling the replication of the experiments accurately. This combination of thorough parameter coverage, detailed experimental results and precise recreation instructions makes the above paper particularly suitable for an experimental verification study. For this test, 3 identical samples were printed with the Ultimaker 2+ FFF printer by using the UltiMaker PLA Green (NFC) filament spool. Both from Ultimaker B.V, sourced from Utrecht, The Netherlands. The samples were printed with the same parameters and the same geometry as those used in one of the tests carried out in the cited article, with the only difference being the extrusion temperature, which was left at the value shown in Table 1 in this case. This value was chosen since it corresponds to the upper limit of the temperatures recommended by the manufacturer of the filament used for this study; utilizing the same temperature as Gonabadi et al. [24] is not fitting for the chosen PLA material.

The parameters used in the study are listed in Table 2, and the appropriate temperature settings for this material are shown in Table 3. The case chosen among those studied in the article is the one with 75% infill, since it is the one where it is mentioned that the strength was the lowest. Finally, the 3 test samples were printed together, equally spaced on the build plate.

Top, bottom and side layers are crucial to providing strength and a smooth surface finish to printed objects. These layers are exposed and form the top and bottom surfaces of the print, so they are often printed thicker to ensure that they are solid and robust. This added thickness helps to cover any inconsistencies or gaps that might be present in the infill structure, resulting in a more uniform and durable surface [40]. This explains the difference in thickness between regular print layers and these layers. The exact thickness of the side and top layers were chosen from the PLA manufacturer’s recommendations.

**Figure 3:**
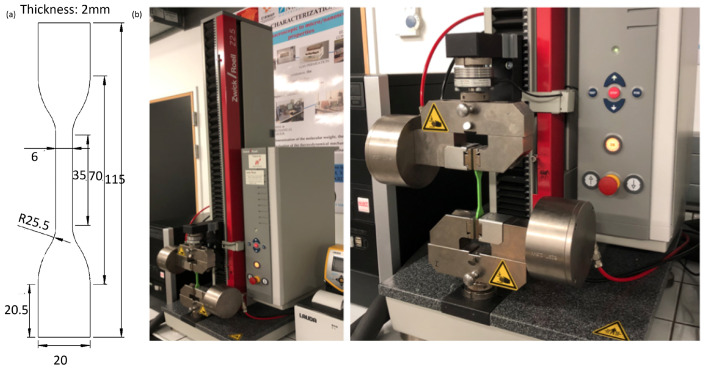
ASTM D638 type IV tensile specimen geometry (**a**) and tensile testing machine (**b**).

The dogbone specimen was designed with the aid of SolidWorks 2020 CAD software and sliced with CURA 4.3, ensuring conformity to the ASTM D638 type IV standard. The specimen dimensions are shown in Figure 3a. The geometry is divided into 2 zones in CURA: the central test zone to which the parameter changes were applied and the pinched edge zones which were printed with 100% infill. Once printed, the specimens were placed in the German Zwick/Roell 72.5 tensile testing machine, manufactured by ZwickRoell GmbH & Co. KG. sourced from Ulm, Germany. The machine provides a Xforce of 2500 N load cell and Type 8297 pneumatic grips from the same manufacturer. The parameters for the tensile experiments are shown in Table 4 The machine is also shown in Figure 3b.

**Figure 4:**
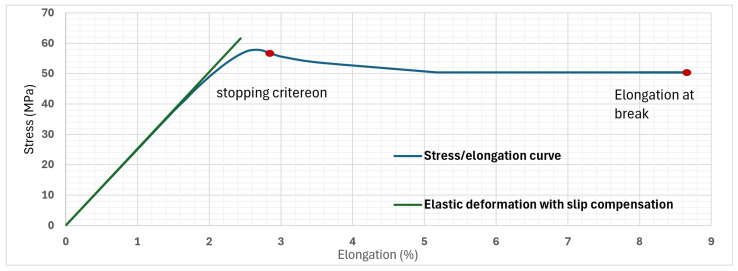
Comparison of Young’s modulus estimates and visualization of elongation at break values.

The tensile testing machine allows yield strength, elongation at break-point and Young’s modulus to be extracted from the output data. To obtain the most accurate Young’s modulus, two values were calculated, accounting and compensating for slip [41]. The slope is shown in Figure 4. All the results can be verified at 75% infill by consulting the previously mentioned study, Gonabadi et al. [24] (see Table 5).

The yield strength value obtained is higher than the one mentioned in the study [24], while the Young’s modulus is comparable. Regarding the elongation at the break point, it cannot be compared because the stopping criterion, which is a predefined condition that determines when the test is terminated, is unknown. On the other hand, in order to obtain results, an estimation of the stopping criterion had to be imposed on the measurements to obtain the same value as the study. The stopping criterion chosen to achieve this was to stop the experiment for a value of stress approximately 4% lower than the maximum measured stress. This value was found by incrementally reducing the stopping criterion until the resulting values matched the study. This gives a very similar result, as shown in Figure 4.

**Figure 5:**
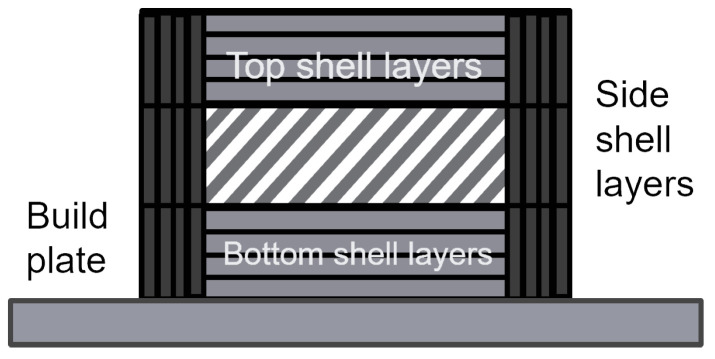
Visualization of print settings.

Unique sets of parameters, visually explained in Figure 5, were set to unique values for each set of experimental test samples. These values can be seen in Table 6. In 8 groups, some experimental variables were set at their maximum or minimum value, and one group was printed with all variables at an intermediate level, which was used to test the linearity hypothesis in the first instance.

Before proceeding with the factorial plan of the experiments, a verification of the consistency between the expected mass with the different infill percentages and the mass actually measured was performed by weighing each sample once on the Adventurer Pro AV264C scale by Ohaus, sourced from Parsippany, NJ, USA, with the resolution = 0.0001 g. As can be seen in Table 7, the results were consistent; therefore, the chosen infill ratio effectively gives the desired material/vacuum ratio.

### 2.2. Factorial Plan of Experiments

After the verification that the experimental setup was valid, the 9 groups of PLA dogbone samples were printed. Each group consisted of 3 specimens with identical printing parameters. This was performed in order to verify the repeatability of the results, which allowed for the verification of the results’ dispersion by calculating the mean and standard deviation for each group.

The tests aimed to determine how the number of top/bottom surface or shell layers, side surface or shell layers and infill ratio affects the mechanical properties of the final printed part. A complete factorial plan was used with preset upper and lower limits for each, the variables of which are detailed in Table 8.

The number of top/bottom surface layers was chosen based on common values found in the literature [42]. Similarly, infill percentage values were chosen based on values commonly employed in industrial part manufacturing [43]. However, very few studies focus on the number of side surface layers; the values were thus chosen based on practical experience. The tests assumed a linear influence between the chosen bounds. Other printing parameters were chosen from the literature study by Gonabadi et al. [24] and are listed in Table 9.

Henceforth, tensile tests on each printed sample were conducted at the speed of 3 mm/min, and the effects of varying each parameter were assessed by extracting the yield strength and Young’s modulus from the resulting stress/strain graphs given by tensile testing software. A linearity verification was conducted to analyze the influence of the three selected parameters (infill percentage, top/bottom thickness and side surface thickness) on the mechanical properties and to see if this influence was linear. Similar work was conducted for the tests intended for the comparison with the literature results.

### 2.3. Numerical Modeling Approach

The numerical modeling of the tensile properties to be predicted was chosen under the assumption that these properties vary linearly with the printing parameters [44]. The first measured value chosen to create a numerical model was the yield strength. Thanks to the set of parameters/yield strength pairs obtained experimentally, the coefficients of the following polynomial can be determined:

(1) $$Y=a_{0}+a_{1}*X_{1}+a_{2}*X_{2}+a_{3}*X_{3}+a_{4}*X_{1}X_{2}+a_{5}*X_{2}X_{3}+a_{6}*X_{1}X_{3}+a_{7}*X_{1}X_{2}X_{3}$$

where the following apply:

— Y, the yield strength (MPa);
— X1, the number of layers of the side surfaces;
— X2, the number of layers of the upper and lower surfaces;
— X3, the infill percentage (%);
— $a_{n}$ (n = 0,…,7), the coefficients of the polynomial.

The coefficients were calculated from the 8 experiments (sample groups 2 to 9), presenting all the combinations of the 3 studied parameters. Experimental group 1, where all the parameters were taken at their central value, was used as reference to see if the choice of linear influence was correct. This is the case if the model is of good quality and the hypothesis of linear influence between the printing parameters and the yield strength is verified.

Similarly, another polynomial was established. This time, the goal was to show a linear correlation between the various printing parameters and the obtained Young’s modulus.

## 3. Results and Discussion

### 3.1. Numerical Model Results

The coefficients obtained for the polynomial linking the mechanical yield strength to the three chosen parameters are shown in Table 10. The table also shows the relative coefficients $a_{1}r$ to $a_{7}r$, which helps determine the significance of each coefficient on the overall calculated mechanical property. A minimal threshold for significance, chosen as 0.05 (5%), helps determine the necessary coefficients to obtain a sufficiently accurate model later.

The calculated coefficients provide detailed insights into how different parameters influence the material’s yield strength. The coefficient $a_{0}=37.12$ represents the baseline yield strength when all predictors (X1, X2, X3) are set to zero. This intercept value indicates that the yield strength starts at 37.12 MPa under these conditions. When comparing this to the observed baseline yield strength of 35.22 MPa, it is quite close to $a_{0}$, with a reduction of 5.11%. This supports the validity of the linear model assumption, showing that the model’s baseline prediction is consistent with the experimental observations. The coefficient $a_{1}=4.56$ signifies that for each additional layer in the side surfaces (X1), the yield strength increases by 4.56 MPa. This coefficient indicates that the number of layers of the side surfaces has a significant impact on enhancing the yield strength, although it is less influential compared with other parameters. The coefficient $a_{2}=9.12$ denotes that for each additional layer in the upper and lower surfaces (X2), the yield strength increases by 9.12 MPa. This coefficient is notably higher than $a_{1}$, suggesting that the number of layers of the upper and lower surfaces has a more pronounced effect on yield strength compared with the number of layers of the side surfaces. Finally, the coefficient $a_{3}=2.77$ implies that each unit increase in the infill percentage (X3) corresponds to a 2.77 MPa increase in the yield strength. Although this coefficient is positive, it is lower than both $a_{1}$ and $a_{2}$, indicating that the infill percentage has a smaller impact on yield strength relative to the number of layers of the upper and lower surfaces and of the side surfaces. Overall, the coefficients reveal that the number of layers of the upper and lower surfaces (X2) has the greatest influence on yield strength, followed by the number of layers of the side surfaces (X1), and finally, the infill percentage (X3). The previous comments do not account for the presence of variables within interaction terms, which further complicates the impact that these individual parameters have on the performance of the material. The coefficients $a_{4}$, $a_{5}$, $a_{6}$ and $a_{7}$ describe interactions between parameters and their effects on yield strength. The negative coefficients $a_{4}=-1.746$ MPa, $a_{5}=-1.17$ MPa and $a_{6}=-1.29$ MPa indicate that the interactions between ($X_{1}$)/($X_{2}$), ($X_{2}$)/($X_{3}$) and ($X_{1}$)/($X_{3}$), respectively, result in a small reduction in the two variables’ collective effect on the yield strength of the samples. Finally, the very small positive coefficient $a_{7}=0.0005$ MPa indicates that the combined optimization of all three parameters results in a miniscule increase in yield strength; this coefficient is non-significant, however, due to its miniscule impact compared with $a_{0}$. These findings highlight complex interactions and a minor combined effect of these parameters on yield strength.

Similarly, the coefficients obtained for the polynomial linking the Young’s modulus to the three chosen parameters are shown in Table 11. Similar work was then conducted to compare the experimental results of Young’s modulus and the numerical model results by utilizing the coefficients shown in Table 11.

The coefficient $a_{0}=1997.02$ MPa represents the baseline Young’s modulus when all predictors ($X_{1}$, $X_{2}$, $X_{3}$) are set to zero. This intercept value indicates that the Young’s modulus starts at 1997.02 MPa under these conditions. This non-zero baseline supports the validity of the linear model assumption, suggesting that the model’s prediction aligns with expected values even in the absence of the other parameters. The coefficient $a_{1}=132.12$ MPa signifies that for each additional layer in the side surfaces ($X_{1}$), the Young’s modulus increases by 132.12 MPa. This coefficient indicates that while the number of layers of the side surfaces positively impacts the Young’s modulus, its effect is moderate compared with other parameters. Meanwhile, $a_{2}=277.82$ MPa denotes that for each additional layer in the upper and lower surfaces ($X_{2}$), the Young’s modulus increases by 277.82 MPa. This coefficient is notably higher than $a_{1}$, suggesting that the number of layers of the upper and lower surfaces has a more pronounced effect on the Young’s modulus compared with the number of layers of the side surfaces. Finally, the coefficient $a_{3}=147.93$ MPa implies that each unit increase in the infill percentage ($X_{3}$) corresponds to a 147.93 MPa increase in the Young’s modulus. Although this coefficient is positive, it is lower than $a_{2}$, indicating that the infill percentage has a smaller impact on Young’s modulus relative to the number of layers of the upper and lower surfaces but still contributes significantly. Overall, the coefficients reveal that the number of layers of the upper and lower surfaces ($X_{2}$) has the greatest influence on Young’s modulus, followed by the infill percentage ($X_{3}$), and then the number of layers of the side surfaces ($X_{1}$). The coefficients $a_{4}$, $a_{5}$, $a_{6}$ and $a_{7}$ describe the interactions between parameters and their effects on the Young’s modulus. The coefficients $a_{4}=-68.79$ MPa, $a_{5}=-58.07$ MPa and $a_{6}=-48.23$ MPa indicate that the interactions between ($X_{1}$)/($X_{2}$), ($X_{2}$)/($X_{3}$), and ($X_{1}$)/$X_{3}$, respectively, impact negatively the two variables’ collective effect on the Young’s modulus of the samples, albeit to a smaller extent. Finally, $a_{7}=9.76$ MPa reflects the three-way interaction among $X_{1}$, $X_{2}$ and $X_{3}$. The positive value suggests that optimizing all three parameters together leads to a modest increase in the Young’s modulus, indicating that the combined effect of these factors is slightly more beneficial than the sum of their individual values and interactions in pairs.

For the yield strength prediction model, the obtained values are shown in Table 12. The table also includes a variant of the predictions where only the coefficients related to the terms without interactions are taken into account. Interaction terms are coefficients affecting more than one variable. The polynomial without interaction terms becomes the following:

(2) $$Y=a_{0}+a_{1}*X_{1}+a_{2}*X_{2}+a_{3}*X_{3}$$

where the following apply:

— Y, the yield strength (MPa);
— X1, the number of layers of the side surfaces;
— X2, the number of layers of the upper and lower surfaces;
— X3, the infill percentage (%);
— $a_{n}$ (n = 0,…,3), the coefficients of the polynomial.

**Table 12:** Comparison of measured yield strengths and numerical model yield strengths.

Sample Group ID	Measured Mean Yield Strength [MPa]	Measurement Range (Maximum Value–Minimum Value) [MPa]	Predicted Yield Strength Redefined without Interactions Terms [MPa]
Sample group 1	35.225	2.345	37.127
Sample group 2	49.370	0.677	53.590
Sample group 3	16.443	0.906	20.664
Sample group 4	36.974	0.102	35.342
Sample group 5	26.93	0.376	26.20
Sample group 6	48.77	0.171	48.04
Sample group 7	40.54	0.177	38.91
Sample group 8	31.66	0.113	29.80
Sample group 9	46.31	0.951	44.45

**Figure 6:**
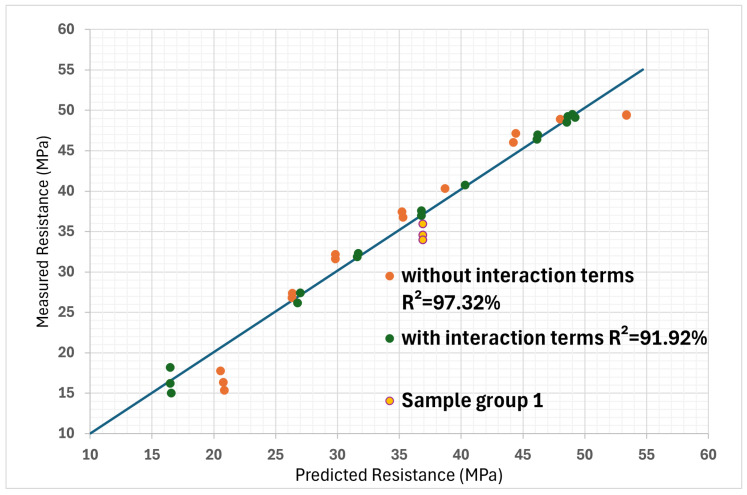
Linearity verification between predicted yield strength values and measured yield strength values, with and without interaction terms. (The line represents a perfect linearity slope, where R^2^ = 100%).

When there is an interaction term, the effect of one variable that forms the interaction depends on the level of the other variable in the interaction. These values help illustrate the individual impact of each isolated printing parameter on the tensile properties of the part. It can be observed that for the case where all the coefficients are taken into account, the values are equal to those of the experiments, which is logical because eight experiments were used to establish eight coefficients. In the case where the interaction terms are neglected, the predictions are a little different but remain rather close to reality. These results are better represented visually in Figure 6. The diagonal line shows perfectly linear results, where R^2^ = 100%. This helps to quickly visually assess the accuracy of the model.

To determine if multicollinearity was present among the predictor variables $X_{1}$, $X_{2}$ and $X_{3}$, the correlation matrix of these predictors was first examined. This matrix revealed that the pairwise correlations among the predictors were low, suggesting that no strong linear relationships existed between them. Additionally, the Variance Inflation Factors (VIFs) for each predictor were calculated. Since the VIF values were found to be close to 1, it indicated that the predictors were not inflating the variance of the regression coefficients significantly. Consequently, based on both the correlation analysis and VIF results, the conclusion was made that there was no significant multicollinearity among the predictor variables.

The probability values obtained for Fisher’s test are shown in Table 13. The probability in % obtained (*p*-value) represents the probability that the influence of the parameter on the result is random. It is generally considered that a *p*-value above 5% indicates that the parameter does not have a significant influence. The *p*-value gives the probability of rejecting the null hypothesis, not the relative importance of the effects. In this case, the hypothesis is that the variation in the parameter impacts the part’s mechanical properties. To evaluate the assumption of the nonexistent influence of the infill ratio on yield strength, calculations were performed as if the infill parameter was not involved. Each measured strength value was multiplied by the total cross-sectional area of the test specimen and then divided by the cross-sectional area of the outer surfaces. The obtained *p*-values, shown in Table 13, confirm the lack of influence that the infill percentage had on the yield strength of the samples; thus, it could safely be eliminated.

**Figure 7:**
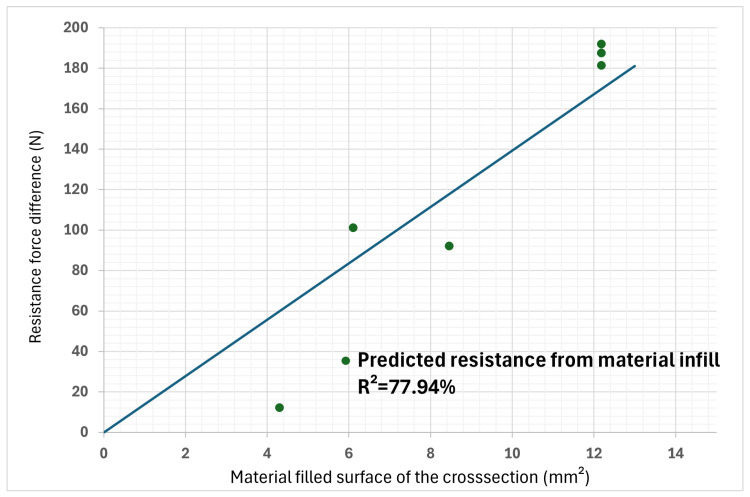
Influence of section infill on yield strength of part. (The line represents a perfect linearity slope, where R^2^ = 100%).

An additional analysis was conducted to verify that the influence of the infill ratio parameter had a linear relation with the yield strength. This analysis was based on the fact that some specimens had the same number of layers in the top/bottom and side surfaces; these were, therefore, differentiated only by the change in the infill ratio. The difference between the maximum force at 75% and at 25% infill was performed for each pair of specimens of the same geometry. This difference in force is attributable only to this parameter. By plotting these differences as a function of the cross-section occupied by the infill, a check on the influence of the cross-section on the properties can be made to see if it is linear. The result of these operations is shown in Figure 7. It can be seen from this graph that the hypothesis of linear influence is plausible but unlikely, since the values fall far from the regression line. The correlation coefficient between the line and the points is equal to R^2^ = 77.94%, which indicates that the infill ratio still has an impact on the yield strength of the printed part, despite it being less significant than the other studied parameters.

**Figure 8:**
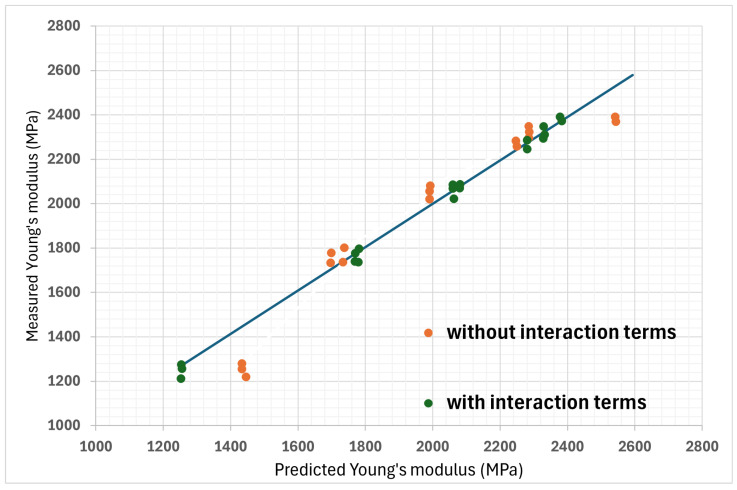
Linearity verification between predicted Young’s modulus values and measured values, with and without interaction terms. (The line represents a perfect linearity slope, where R^2^ = 100%).

To establish the coefficients of the polynomial linking the Young’s modulus to the three chosen parameters, coefficients were calculated as previously. For the Young’s modulus prediction model, a linearity verification was conducted with and without the interaction terms, and the difference is plotted in Figure 8. Fisher’s test was also performed on the Young’s modulus results. The *p*-values obtained are shown in Table 14. It can be seen that the order of importance is different from what was deduced from the previous yield strength model. The thickness of the upper and lower surfaces clearly has a significant influence. The infill ratio is below the redundancy value but close to it, and finally, the thickness of the side surfaces is not considered to have a significant influence on the Young’s modulus.

### 3.2. Simplified Section Area Numerical Model

**Figure 9:**
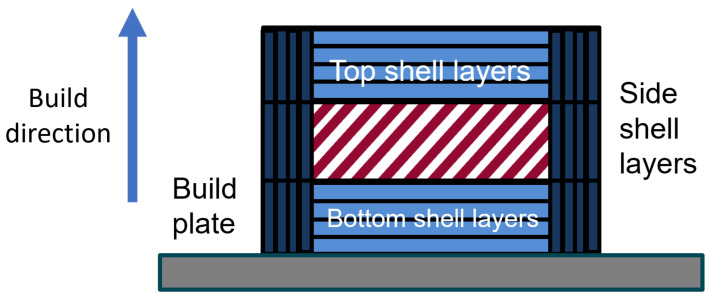
The cross-section of a part generated by the slicer.

Since the results of Fisher’s test indicate that the infill percentage could be ignored only for the yield strength prediction model, a new model was considered. The idea is to reconstruct the model with the proportion of the different zones in the tested cross-section instead of the number of layers of the side and top/bottom surfaces. The two zones considered were the side surface parts in dark blue in Figure 9 and the top and bottom layers in lighter blue in the same figure; this model completely eliminates the infill percentage from the calculations.

The two new parameters chosen are, therefore, the proportion in % of the side surfaces and the percentage of the lower and upper layers in the total section of the specimen. In Table 15, it can be seen that the values of the designations vary between 0 and 4, with each value representing an exact sidewall or top/bottom wall percentage. These new parameters were used to obtain new coefficients for a new polynomial with the same method as before. The coefficients obtained with the same polynomial method as the yield strength without interaction are shown in Table 16.

**Figure 10:**
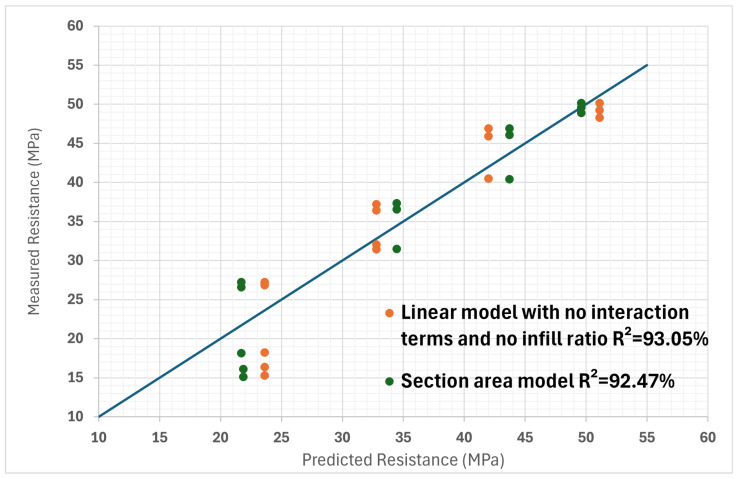
Comparison of linear model values without infill and without interaction terms and section area model. (The line represents a perfect linearity slope, where R^2^ = 100%).

The results are also shown in Figure 10, which shows the old predictions without interaction and without the infill percentage coefficient in order to evaluate the efficiency of the section area model. It can be seen that the point couples appear to be better distributed on either side of the slope line with the new model. This is confirmed by a calculation of the correlation coefficient in both cases between the measurements and the model predictions. The original model has a value of R^2^ = 87.95%, while the new model reaches R^2^ = 89.57%. The yield strength of the sample where all the parameters are at their median value, however, is not as accurately predicted as before.

**Figure 11:**
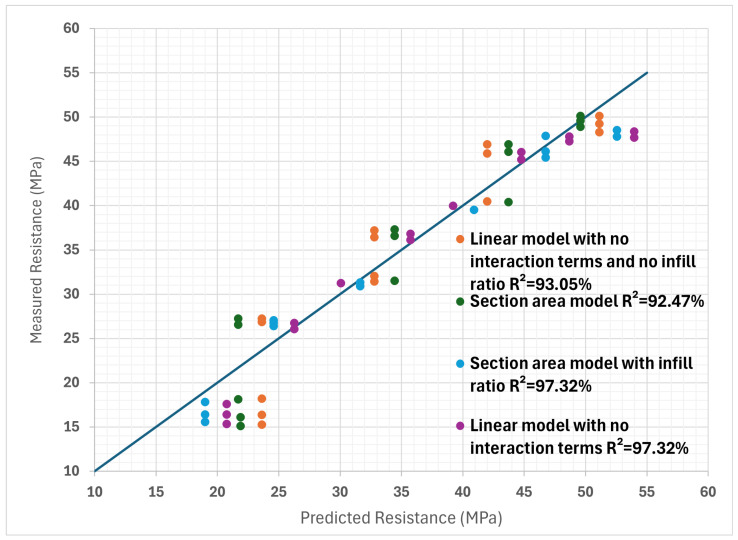
Comparison of linear model values without infill and without interaction terms, section area model, linear model without interaction terms but with infill and section area model with infill considered. (The line represents a perfect linearity slope, where R^2^ = 100%).

A possible improvement to this model would be to also take into account the infill percentage, which was omitted in the section area model. Even if its influence has been identified as being less important, improvements to the model can potentially be found. By using the same approach as before but with infill percentage in addition, new coefficients can be determined for the polynomial. Through a calculation of the correlation coefficient between the measurements and the model predictions for the original model without interactions and the area model with the infill percentage, it can be seen that the area model is again slightly better. The original model has a value of R^2^ = 92.44%, while the new model reaches R^2^ = 96.06%. It can be seen that all the possibilities of the models summarized so far in Figure 11.

A second series of tests was conducted. The new experiments were performed on samples with different parameters from the first set of tests, both within and outside the previous bounds. The validity of the models predicting yield strength was evaluated.

## 4. Numerical Model Result Summary

Various numerical models, in the form of polynomials, were employed to assess the relationship between print settings and the yield strength. Initially, the complete polynomial numerical model incorporating interaction terms exhibited good potential for predicting the yield strength from the print settings, quantified by an R^2^ value of 91.92%. Subsequently, by simplifying the model and removing the interaction terms, an even higher level of agreement between the predicted and observed results was achieved, as evidenced by the improved R^2^ value of 97.32%. Moreover, to evaluate the impact of omitting infill percentage on the analysis of the yield strength model, the infill data were excluded from the simplified polynomial model, resulting in a slightly reduced yet promising R^2^ value of 93.05%. Additionally, a section area model, an alternative approach employed, demonstrated a favorable level of fit with the observed data, attaining an R^2^ value of 92.47%. Finally, incorporating the infill percentage into the section area model attested to its effectiveness, as it resulted in the R^2^ value of 97.32%. The results are summarized in Table 17.

When all tests are evaluated, with all the parameters taken into account, the section area model is a better predictor of yield strength, as it has a higher correlation coefficient. The agreement between the predictions of the various models and the measurements can also be assessed visually in Figure 11. Here, it can be seen that all models have difficulty in estimating the strength of some specimens, since they are far from the perfect linear correlation line. It is possible that the assumption of linearity is wrong for the infill percentage and that it has a weaker influence on yield strength at lower percentages.

## 5. Conclusions and Perspectives

Additive manufacturing is rapidly expanding, and the need to be able to evaluate the properties of the produced parts from the parameters used for printing is crucial to saving time and resources. The objective of this work was to determine the influence of three selected parameters on the mechanical properties of manufactured parts by FFF and to propose a model for their evaluation. This was performed by exploring the effect of varying material infill, number of layers for top/bottom surfaces and number of layers for side surfaces on the mechanical properties of the printed parts. The number of layers of the top/bottom surfaces and the number of layers of the side surfaces are not well explored in the literature. Furthermore, within the scope of this paper, the overarching goal was to develop a predictive model capable of anticipating the tensile properties of parts, calculated directly from their print settings. The study also helped highlight the linear correlation between these parameters and the yield strength of the printed parts.

The following are the conclusions extracted from this study:

- Concerning yield strength, the number of layers of the upper and lower surfaces is the most important parameter, followed by the number of layers of the side surfaces; finally, the infill ratio is the least important parameter.
- Young’s modulus is the most influenced by the number of layers of the upper and lower surfaces, then by the infill ratio and finally by the number of layers of the side surfaces.
- A mathematical model for the prediction of yield strength was developed. This model links the three parameters studied with the strength via a polynomial; the coefficients were determined experimentally.
- Another model based on the fractions of the area of the different homogeneous parts in a section of a 3D-printed part was created. The results obtained were closer to the experimental results than those of the first model with the same number of coefficients.
- Both models were evaluated on new specimens inside and outside of the initial print setting ranges, and the overall the results align well with the predictions.
- The presence of interaction terms indicates that the effect of one printing parameter varies according to the value of another. Simplifying the polynomials for both models by eliminating the interaction terms showed that their impact on the model’s accuracy is minimal.
- Given that the *p*-value of the infill percentage’s impact on yield strength is the highest, it has the highest likelihood of rejecting the null hypothesis of the test; thus, a decision was made to eliminate it from models predicting yield strength only and the simplified section area model.
- Similarly, for Young’s modulus, the *p*-value obtained from the number of layers of the side surfaces shows that it can also be eliminated, but at a higher detriment than the infill percentage in the first case.

## Figures and Tables

**Table 1:** Printing parameters for literature result replication from Gonabadi et al. [24].

Regular Layer Thickness	T° Extrusion	T° Plate	Print Speed	Material
0.15 mm	210 °C	60 °C	60 mm/s	PLA

**Table 2:** Various printing parameters for the dogbone specimen.

Print Nozzle	Top/Bottom Layer Thickness	Side Surface Thickness	Infill Method	Extrusion Speed
0.4 mm	0.9 mm	1.2 mm	Grid	2 mm/s

**Table 3:** Temperatures used for the printing of validation PLA samples and expected fracture limit and Young’s modulus values according to the PLA’s manufacturer.

Printing T (°C)	Plate T (°C)	Max T (°C)	Fracture Limit (MPa)	Young’s Modulus (MPa)
180–230	45–60	50–60	65	3000–3500

**Table 4:** Parameters for the tensile experiments.

Loading Direction	Applied Force (N)	Tensile Test Speed (mm/min)
Unidirectional along length of sample	2500	3

**Table 5:** Comparison of results obtained through experimental testing with those of study [24].

Properties	Yield Strength (MPa)	Young’s Modulus (MPa)	Elongation at Break Point %
Gonabadi et al. [24]	36±0.5	2500±50	2.85±0.15
Mean value	56±2	2599±28	8.68±1.25

**Table 6:** List of test group printing parameters.

Sample Group ID	Parameter Designation	Side Surface Layers	Top/Bottom Layers	Infill Percentage
Sample group 1	0 0 0	2	4	50
Sample group 2	1 1 1	3	6	75
Sample group 3	−1 −1 −1	1	2	25
Sample group 4	1 −1 1	3	2	75
Sample group 5	−1 −1 1	1	2	75
Sample group 6	1 1 −1	3	6	25
Sample group 7	−1 1 −1	1	6	25
Sample group 8	1 −1 −1	3	2	25
Sample group 9	−1 1 1	1	6	75

**Table 7:** Comparison of expected masses and average of measured masses to verify infill percentage.

Sample Group ID	Expected Mass [g]	Mean [g]	Difference [g]	Relative Difference [%]
Sample group 1	5.18	5.11±0.05	−0.07	−1.4
Sample group 2	5.48	5.43±0.05	−0.05	−0.98
Sample group 3	4.61	4.63±0.02	0.021	0.46
Sample group 4	5.37	5.36±0.04	−0.01	−0.24
Sample group 5	5.26	5.23±0.02	−0.03	−0.57
Sample group 6	5.26	5.30±0.01	0.04	0.76
Sample group 7	5.10	5.02±0.01	−0.07	−1.54
Sample group 8	4.93	4.91±0.01	−0.01	−0.34
Sample group 9	5.43	5.31±0.01	−0.11	−2.13

**Table 8:** Factorial plan print settings.

Designation	−1	0	1
Number of side surface layers (X1)	1	2	3
Number top/bottom layers (X2)	2	4	6
Infill percentage (%) (X3)	25	50	75

**Table 9:** Factorial plan fixed printing parameters.

Printing T (°C)	Plate T (°C)	Print Speed	Print Nozzle	Infill Method	Material	Layer Thickness
210	60	60 mm/s	0.4 mm	grid	PLA	0.15 mm

**Table 10:** Yield strength polynomial coefficients.

$a_{0}$	$a_{1}$	$a_{2}$	$a_{3}$	$a_{4}$	$a_{5}$	$a_{6}$	$a_{7}$
37.12	4.56	9.12	2.77	−1.746	−1.17	−1.29	0.0005
$a_{0}$/$a_{0}$	$a_{1}$/$a_{0}$	$a_{2}$/$a_{0}$	$a_{3}$/$a_{0}$	$a_{4}$/$a_{0}$	$a_{5}$/$a_{0}$	$a_{6}$/$a_{0}$	$a_{7}$/$a_{0}$
1	0.122	0.139	0.074	−0.047	−0.02	−0.034	0.00001

**Table 11:** Young’s modulus polynomial coefficients.

$a_{0}$	$a_{1}$	$a_{2}$	$a_{3}$	$a_{4}$	$a_{5}$	$a_{6}$	$a_{7}$
1997.02	132.12	277.82	147.93	−68.79	−58.07	−48.23	9.76
$a_{0}$/$a_{0}$	$a_{1}$/$a_{0}$	$a_{2}$/$a_{0}$	$a_{3}$/$a_{0}$	$a_{4}$/$a_{0}$	$a_{5}$/$a_{0}$	$a_{6}$/$a_{0}$	$a_{7}$/$a_{0}$
1	0.066	0.139	0.074	−0.034	−0.029	−0.024	0.004

**Table 13:** Fisher’s test results for yield strength.

Parameter	*p*-Value
The number of layers of the top/bottom	0.179%
The number of layers of the side surfaces	2.095%
The infill ratio	8.856%

**Table 14:** Fisher’s test results for Young’s modulus.

Parameter	*p*-Value
The number of layers of the top/bottom surfaces	0.563%
The number of layers of the side surfaces	6.161%
The infill ratio	4.484%

**Table 15:** Test parameters for the section area model.

Sample Group ID	Side Surface [%]	Top/Bottom [%]	Side Surface Standardized	Top/Bottom Standardized
Sample group 1	26.66	29.33	0	2
Sample group 2	40	36	1	0
Sample group 3	13.33	17.33	−1	−2
Sample group 4	40	12	1	−1
Sample group 5	13.33	17.33	−1	−2
Sample group 6	40	36	1	0
Sample group 7	13.33	52	−1	1
Sample group 8	40	12	1	−1
Sample group 9	13.33	52	−1	1

**Table 16:** Simplified section area model polynomial coefficients.

$a_{0}$	$a_{1}$	$a_{2}$
38.78	7.89	12.46

**Table 17:** Results of the various models.

Model	R^2^
Initial polynomial with interaction terms	91.92%
Initial polynomial without interaction terms	97.32%
Initial polynomial without interaction terms without infill	93.05%
Section area model	92.47%
Section area model with infill	97.32%

## Data Availability

The original contributions presented in the study are included in the article, further inquiries can be directed to the corresponding author.
